# Application of Portfolio Theory to Healthcare Capacity Management

**DOI:** 10.3390/ijerph18020659

**Published:** 2021-01-14

**Authors:** Carina Fagefors, Björn Lantz

**Affiliations:** 1 Department of Pediatric Anesthesiology, Intensive Care and Neonatology, Sahlgrenska University Hospital, 413 45 Gothenburg, Sweden; carina.fagefors@vgregion.se; 2 Technology Management and Economics, Chalmers University of Technology, 412 96 Gothenburg, Sweden; 3 Department of Engineering Science, University West, 461 86 Trollhättan, Sweden

**Keywords:** portfolio theory, capacity pooling, healthcare management, capacity planning

## Abstract

Healthcare systems worldwide are faced with continuously increasing demand for care, while simultaneously experiencing insufficient capacity and unacceptably long patient waiting times. To improve healthcare access and availability, it is thus necessary to improve capacity utilization and increase the efficiency of existing resource usage. For this, variations in healthcare systems must be managed judiciously, and one solution is to apply a capacity pooling approach. A capacity pool is a general, collaborative capacity that can be allocated to parts of the system where the existing workload and demand for capacity are unusually high. In this study, we investigate how basic mean-variance methodology from portfolio theory can be applied as a capacity pooling approach to healthcare systems. A numerical example based on fictitious data is used to illustrate the theoretical value of using a portfolio approach in a capacity pooling context. The example shows that there are opportunities to use capacity more efficiently and increase service levels, given the same capacity, and that a mean-variance analysis could be performed to theoretically dimension the most efficient pooling organization. The study concludes with a discussion regarding the practical usefulness of this methodology in the healthcare context.

## 1. Introduction

Capacity management in service operations can be challenging, as the production and consumption of the provided service must be executed simultaneously [1]. In healthcare services, these issues gain prominence as the failure to meet demand can have serious consequences and, in a worst-case scenario, result in fatalities [2,3]. Healthcare systems in Sweden are experiencing scarcity of resources along with difficulties in efficiently meeting demand for healthcare, resulting in low accessibility, longer queues, and high workloads [4]. Numerous reports further indicate that access to healthcare personnel will remain at existing levels for the foreseeable future [4,5], while the need for care will increase during the same period [6]. Hence, the lack or paucity of resources that healthcare organizations are experiencing is unlikely to improve without a change in capacity management practices.

The match between capacity and demand in service operations is further impeded by the existence of random variations in a system [7]. In healthcare systems, sick leave and vacancies are common reasons for variations in available capacity, while patient arrival and length of stay are common causes of the variations in healthcare demand [8]. The planning process becomes even more challenging in healthcare organizations, where a complex network of facilities, equipment, and trained workforce must be coordinated [9]. In Sweden, capacity management is often decentralized in specific units or departments within a healthcare organization, and those units are responsible for managing the short-term variations in capacity by applying different flexibility solutions, such as overtime work and the queueing of patients [10,11,12]. However, these flexibility solutions are expensive and may cause other issues such as an impaired work environment for personnel and a lack of patient safety [13]. A change in healthcare capacity management strategy is thus necessary to match the available resources with healthcare demand more efficiently.

Decentralized capacity management in tandem with variations in capacity and demand can create a shortage of resources at the operational level of the units and departments, while the capacity of the system as a whole may simultaneously be sufficient. For example, one unit can experience a lack of capacity due to short-term leave, while another unit can simultaneously experience excess capacity. Short-term flexibility solutions must thus be applied at the decentralized planning level to provide the required capacity, while the capacity of the entire system is considered capable of fulfilling it. Hence, on the aggregate level of a system, the effect of variations in capacity due to random causes is often reduced [14]. Therefore, if, to some extent, capacity allocation could be performed at a higher level in the organization, the effects of variations could be curtailed, and resources could be used more efficiently.

One tool for exploiting the benefits of aggregated capacity allocation and achieving a more efficient use of resources is a capacity pool, which is a general capacity that can be allocated to parts of the system where the short-term need for resources is unusually high [3,15,16]. Capacity pools have frequently been applied within manufacturing industries and can be used to successfully increase capacity utilization [17]. Capacity pools have also occasionally been implemented within the healthcare sector, but there is limited research on its impact on capacity utilization. The literature on the topic is weak and largely anecdotal, directed mostly toward pools of nurses [18,19,20,21]. These studies indicate that implementing a capacity pooling approach is wrought with difficulties, like the necessity of introduction and training programs to secure the work environment and ensure patient safety. However, there are several theoretical advantages to implementing capacity pools [15,16,17,22]. For example, the average waiting time can be reduced when there is a single queue to all units, rather than separate queues for different units. Furthermore, a higher capacity utilization can be achieved with the same or higher service level.

A mathematical framework that can be applied to this dimension and optimize a capacity pooling approach is the mean-variance analysis used in modern portfolio theory. Portfolio theory was first introduced by Markowitz in the finance sector in 1952 and aims to maximize the expected return on investments for a given level of risk [23,24]. A portfolio consists of several assets whose risk and return should be considered collectively, instead of individually. The overall risk of a portfolio can be reduced through diversification, when the returns and risks of the included assets are not perfectly synchronized. A fundamental component of portfolio theory is, therefore, used in the capacity pooling approach; in that, when several sources of variations are aggregated, the total effect of the variability can be reduced [14].

Portfolio theory has been creatively applied in other areas outside the finance sector, such as electricity planning, psychology, biosecurity, and operations management studies [25,26,27,28,29]. For example, Cardazo and Smith [25] conducted an exploratory empirical study in the early 1980s in which they found that the mathematical framework of portfolio theory could be applied to design and used to manage a company’s portfolio of products and services, with implications for the allocation of the organization’s resources. The framework has also been applied in the electricity sector to optimize electricity planning [27,28,29], as well as within the biosecurity sector to efficiently allocate surveillance efforts [30,31]. Although the empirical setting varies, the conclusion of numerous studies is that the mean-variance analysis framework in portfolio theory can be used to allocate scarce resources to parts of the system where they are most required [30,31,32]. However, to the best of our knowledge, portfolio theory has not been applied to a healthcare setting to optimize the allocation of resources, where the characteristics of capacity management differ starkly from other fields.

In this study, we investigate how the basic mean-variance methodology from portfolio theory can be applied as a capacity pooling approach to healthcare systems. The paper is structured as follows. In Section 2, the statistical framework is outlined, followed by Section 3, where a fictitious case is provided and its results are obtained. The results are discussed in Section 4, followed by a conclusion in Section 5.

The paper contributes to the literature by illustrating and emphasizing the theoretical value of pooling approaches in healthcare capacity management. Although there is a substantial theoretical upside with the pooling approach, we also show that the allocation of units to pools is a complex issue. Hence, simulation approaches are probably required to achieve efficient pooling structures in more complex healthcare systems. In addition, there are of course many other practical issues related to the rationalizing of healthcare resources to face the increasing complexity of the demand of services in relation to which capacity pooling approaches need to be analyzed.

## 2. Materials and Methods

The *expected value* of the sum of two (or any arbitrarily larger number of) random variables is simply the sum of their individual expected values [33], regardless of any correlation between them. The variance of the sum of two random variables is, however, equal to the sum of their individual variances if and only if they are independent, or if there is no statistical correlation between them [33]. Hence, if the assumption of independence holds, we may simply write it as follows:

(1) $$\sigma_{P}^{2}=\sigma_{1}^{2}+\sigma_{2}^{2}$$

where $\sigma_{P}^{2}$ is the variance of the sum of the random variables 1 and 2, and $\sigma_{1}^{2}$ and $\sigma_{2}^{2}$ are their individual variances.

When the two random variables are correlated, an adjustment must be made in calculating the variance of their sum, by adding twice their covariance [33]:

(2) $$\sigma_{P}^{2}=\sigma_{1}^{2}+\sigma_{2}^{2}+2\sigma_{1,2}^{2}$$

where $\sigma_{1,2}^{2}$ is the covariance between the random variables 1 and 2. Hence, Equation (1) is simply a special case of Equation (2), where there is no covariance between the two random variables.

As the covariance between two random variables can statistically be defined as the product of three factors, the standard deviations of the respective random variables, and their (Pearson) correlation coefficients [33], Equation (2) may also be written as follows:

(3) $$\sigma_{P}^{2}=\sigma_{1}^{2}+\sigma_{2}^{2}+2\sigma_{1}\sigma_{2}\rho_{1,2}$$

where $\rho_{1,2}$ is the correlation between random variables 1 and 2.

In the more general case, with *n* random variables, Equation (3) becomes the following [33]:

(4) $$\sigma_{P}^{2}=\sum_{i=1}^{n}\sigma_{i}^{2}+2\sum_{i=1}^{n}\sum_{j>i}^{n}\sigma_{i}\sigma_{j}\rho_{i,j}$$

The statistical relationships in Equations (1)–(4) can be used to create a few simple albeit enlightening theoretical examples of the potential efficiency of capacity pooling in different situations in the service operations context of a healthcare system. Assuming that a certain service system comprises two isolated units, where the expected daily capacity in units one and two are 40 workers with a standard deviation of six workers and 30 workers with a standard deviation of four workers, respectively (“workers” can be replaced with any other unit for capacity measurement). It is also assumed that the management has decided that a safety capacity corresponding to one standard deviation should be kept in each unit, so that unit one is staffed daily with 46 workers and unit two with 34 workers. If the variation in the required capacity can be approximated by a normal distribution, the service level in each unit is approximately 84%.

If the actual daily capacity requirements in the two units are independent, that is, if there is no correlation between them, a simple pooling approach would yield an expected daily capacity of 70 workers. An application of Equation (1) shows that the standard deviation in the expected daily capacity of the pool is 7.2 workers. This corresponds to an increase in service levels to approximately 92%. In this case, the pooling approach increased service levels significantly, without adding extra capacity to the system as a whole.

Now, assume that the daily capacity requirement of the two units is positively correlated. In other words, in periods when the required capacity in unit one is high, there is a tendency for the required capacity in the second unit to be high too. If the correlation coefficient is, for example, 0.3, application of Equation (3) shows that the standard deviation in the daily required capacity of the pool is 8.1 workers. In this case, the pooling approach would increase the service level from 84% to 89%. Similarly, a correlation coefficient of 0.7 would yield a service level of 86%. Although a positive correlation between the capacity requirements of the two units decreases efficiency, using the pooling approach is still beneficial. In the extreme (but typically unrealistic) case where there is a perfect positive correlation, there would be no theoretical benefit (but no harm either) in using a pooling approach.

We now turn to examine the case when the correlation is negative, that is, if there is a tendency for the required capacity in unit two to be high when the required capacity in unit one is low. If the correlation coefficient is, for example, −0.2, application of Equation (3) would show a standard deviation of 6.5 workers in the expected, required daily capacity of the pool, corresponding with an increase in service level from 84% to 94%. Similarly, a correlation coefficient of −0.5 would yield a service level of 97%. Thus, a negative correlation between the capacity requirement of the two units reinforces efficiency in the pooling approach, and it is easy to see that the effect becomes stronger for lower correlations. In the extreme (but generally unrealistic) case where there is a perfect negative correlation, the standard deviation in the expected required capacity of the pool would be 2 workers, corresponding to a service level very close to 100%.

## 3. Results

(The numerical data for this fictitious case were simulated, and can easily be replicated, using the random number generator in the Analysis ToolPak in Excel 365. The following configuration can be used: number of variables = 5, number of random numbers = 100, distribution = Poisson, lambda = 30, random seed = 111. Other configurations will of-course yield minor, but immaterial deviations from the numerical results presented here.) Suppose that a healthcare department has five units in a large city. The department management believes that each unit has an average of 30 patients per day, with an equal likelihood of upward and downward variations. Currently, each unit is adequately staffed to provide service for 30 patients; hence, each unit has a service level of approximately 50% if the variation in the daily demand approximates a normal distribution. To increase service levels, it is decided to add safety capacity so that the staffing at each unit can handle patient numbers equaling its expected daily demand plus one standard deviation. This would increase the service level to approximately 84%.

Statistics (assumed to be representative) from the last 100 days reflecting daily demand in terms of the number of patients in each unit can be seen in Table 1 below. Therefore, unit one, for example, should be staffed enough to provide care for 29.4 + 5.0 = 34.4 patients per day (for the sake of argument, we assume that a patient can be partly served). The total capacity in the hospital department as a whole would be 177.4 patients per day.

However, the hospital department also considers pooling its capacity, including the safety capacity mentioned above. From the data collection described above, the company was able to calculate the correlations in demand between all pairs of units (see Table 2 below). For managerial and other reasons, all five units cannot comprise a single pool, and every unit must be included in a pool with at least one other unit. Hence, two pools should be created, with three and two units, respectively. However, the question remains as to how the units should be allocated to the pools and whether the manner of allocation actually matters.

There are ten ways to select three elements from a set of five; hence, there are ten possible pool configurations in this situation. Table 3 below shows the variances for the pools in different configurations, calculated based on Equation (4), and the resulting service levels. It is apparent that pooling increases service levels throughout, from the original 84%. However, some configurations are clearly better than others. Configuration 8 seems to be the worst, in terms of average service level, while configuration 7 seems to be the best. Hence, how units are formed into pools affects the results.

There are a few major theoretical takeaways from this fictitious case. First, pooling is generally an efficient way to manage capacity. Second, capacity pooling is generally more efficient when the correlation of capacity requirements between the units is low. Third, although having a larger number of units in a pool is more efficient, the marginal effect on service levels is diminishing. Therefore, careful analysis must be performed to find the best pool configuration, especially in situations where there are several possible ways to form capacity pools out of individual units. The best average service level of the pool configuration must be found, given the possible barriers to capacity pooling.

In the next section, these insights will be further discussed.

## 4. Discussion

Pooling is an efficient theoretical means of managing capacity, and a mean-variance analysis can be performed to dimension and organize units into capacity pools. From the example provided here, it was concluded that a higher service level could be achieved with the same capacity, if organized into pools, resulting in higher capacity utilization of the entire system. Several studies argue that capacity pools can be an efficient tool to manage capacity [3,17,18,34], and that the mean-variance framework could be used to allocate and optimize the use of scarce resources [30,31,32]. However, these studies were applied in settings with different conditions from those in the healthcare sector, such as the requirement of simultaneous production and consumption of the provided service [1], and the effect on patient safety if demand cannot be met in due time [2,3]. The fictitious case provided in this study shows that there are opportunities to implement capacity pools in healthcare settings, but further research with real data is needed.

Capacity pooling is generally more efficient when the correlation between the capacity requirements of the units is lower. Hence, if one unit within a period of time requires more capacity than average, while another unit during the same period requires less, the total capacity utilization can be improved if both units are organized within the same capacity pool. The variations in capacity requirements will thus have a limited effect on performance when the allocation of resources is aggregated [3,14]. There are several examples in the healthcare sector of low, or even negative, correlation in capacity requirements between units due to seasonal variations. For example, Schrijver et al. [35] found that the demand for pediatric care due to respiratory tract infections tends to peak in January, while demand for pediatric care due to asthma peaks in March and October. The capacity required to provide healthcare for the two conditions will thus increase during different periods of the year. Hence, there is theoretically a low or even negative correlation between the healthcare demand for these two diseases in this particular example. To achieve a high total capacity utilization, it could thus be beneficial to organize the capacity required to treat these conditions within the same capacity pool. However, while considering inclusion in the same pooling approach, several factors must also be considered, such as the degree of difference in the required professional competence for different conditions.

Using capacity pools can be efficient, even though there is a positive correlation in capacity requirements between units. For example, variations in short-term leave, such as sick leave, are seasonal, with peaks often occurring during winter, and the correlation between units due to seasonal sick leave is, therefore, likely positive. However, as previously stated, when several sources of random variability are aggregated, the relative influence of variability will decrease [14]. Today, short-term leave in healthcare systems is often handled by applying flexibility tools such as overtime, queuing patients, or summoning temporary staff [8,10,36,37,38]. These solutions are often costly, with negative effects on both patient safety and the work environment [38,39]. If instead, a capacity pooling approach is implemented to manage short-term variation in capacity requirements, the use of costly solutions could be reduced, despite the positive correlation in capacity requirement between units allocated to the same capacity pool.

In addition to permitting a more efficient use of resources in the entire system, the capacity pooling approach has other potential benefits too. For example, employees that rotate between departments can enable knowledge exchange between the units, and working in a capacity pool may improve skills and professional development [40]. Furthermore, an efficient match between healthcare demand and capacity reduces waiting times for patients, increases service levels, and enhances patient safety [41,42,43,44].

To efficiently implement a capacity pooling approach, barriers to capacity pooling must be considered. Although a high service level can theoretically be obtained, there might be barriers to the inclusion of specific healthcare units in a pool. For example, healthcare departments are often specialized, and professional competence could be a barrier to sharing capacity in a pooling approach. Moreover, if routines and procedures differ vastly between units, it might be difficult for employees in a capacity pool to rotate between units, resulting in inefficient time usage and patient safety issues [18,21,34]. There are studies that suggest that the work environment in a capacity pool can be stressful and that there are difficulties in recruiting qualified staff to the pools. Cavouras [45] suggested that incentives must be found for recruiting employees to such an organization in order for it to function as intended. The distance between units in a pool, both geographically and organizationally, might be an impediment for the pool to work efficiently. These potential barriers must be considered before organizing units into a capacity pooling system.

Although a larger number of units in a pool is more efficient, the marginal effects on service level diminish with an increased number of units in a pool. Cattani and Schmidt [17] argue that, by including three parties in one capacity pool, the service level will be enhanced, but the effect is not as apparent as when the first two parties are pooled. The marginal effect on the service level of adding one extra unit to the pool might thus be negligible or even negative when potential obstacles to pooling are considered.

The fictitious example provided in this study is of course simplified and theoretical and is based on variability in capacity requirements. However, reality is often more complex, and the variation in capacity requirement should be matched with variations in healthcare demand, as alignment between the two is crucial [41]. As previously mentioned, the matching of capacity and demand is particularly difficult in service operations management, where production and consumption of the service must be executed simultaneously [1]. Real data should thus be used to calculate the potential effects of capacity pools, and is thus an area for future research.

In summary, portfolio theory is a potential candidate for dimensioning and organizing capacity pools in healthcare systems, which will provide opportunities to improve capacity utilization in the healthcare sector. In the fictitious example provided in this study, we saw that many benefits could accrue in terms of capacity utilization and service level by pooling only five different units. The possible consequences for a larger organization with a network of units and departments that utilize a capacity pooling approach could thus be significant. The increased capacity utilization could not only result in shorter waiting times for patients, but also greater work satisfaction and increased patient safety [3,41,42,43,44]. Further research should, therefore, be conducted using real data to calculate possible capacity pool configurations and potential capacity utilization.

## 5. Conclusions

In this study, we have shown how the mean-variance analysis framework used in modern portfolio theory could be applied to optimize the use of capacity and dimension a capacity pooling approach in a healthcare setting. The fictitious example of capacity pooling shows that there are opportunities to use capacity more efficiently and increase service levels, given the same capacity. Moreover, it was shown in the fictitious example that a mean-variance analysis could be performed to theoretically dimension the most efficient pooling organization.

There are several potential benefits of implementing a capacity pooling approach in a healthcare setting. A more efficient match between capacity and demand can reduce waiting times and patient queues [41]. The use of costly short-term flexibility solutions in capacity management such as overtime, queuing patients, and calling temporary staff can be reduced. Moreover, the work environment can be improved when peaks in capacity requirements are mitigated, and patient safety is enhanced.

This study introduces the idea of a systematic application of capacity pooling through portfolio theory in healthcare systems. In practice, there are of course a lot of other issues related to the rationalizing of healthcare resources to face the increasing complexity of the demand of services; however, it would be beyond the scope of this paper to cover all those issues.

As stated above, the example provided in this analysis is performed using fictitious data. The case is a simplified version of reality, where several aspects are not considered. For example, the variability in both healthcare demand and healthcare capacity must be considered, while our given example only provides for the variability in healthcare demand. Moreover, several units might not be suitable, or even possible, to include in the same capacity pool owing to various potential barriers to capacity pooling. Therefore, there are two major implications for future research on capacity pooling in the healthcare setting. Firstly, simulations or even analyses based on real data should be used to capture the complexity of matching capacity and demand in a healthcare setting, and a mean-variance analysis performed to calculate how a capacity pooling approach could be organized. In particular, simulation could be used to deal with the variability of demand and capacity in a way that captures a more realistic view of a healthcare system and to conduct sensitivity analyses of various pooling configurations. Secondly, barriers to implementing capacity pools, such as professional competence and geographical distance, must be further investigated before organizing the units into potential capacity pools. In addition, the potential for capacity pooling in other sectors, or in service operations in general, may also be an effective research topic.

## Figures and Tables

**Table 1:** Daily demand for the five units.

	1	2	3	4	5
Mean	29.4	29.9	31.1	30.1	29.5
Standard deviation	5.0	5.7	4.9	6.2	5.6

**Table 2:** Correlations.

	2	3	4	5
1	−0.085	0.164	0.261	−0.050
2		−0.105	−0.099	0.142
3			0.032	−0.021
4				0.098

**Table 3:** Pool configurations and the resulting service levels.

Configuration	Units in Pool	Total Daily Capacity	Standard Deviation	Service Level
1	1,2,3	106.0	8.9	96.1%
	4,5	71.4	8.7	91.1%
2	1,2,4	106.3	10.0	95.4%
	3,5	71.1	7.4	92.3%
3	1,2,5	105.1	9.5	95.7%
	3,4	72.4	8.1	91.7%
4	1,3,4	106.7	10.7	93.5%
	2,5	70.7	8.5	90.7%
5	1,3,5	105.5	9.2	95.4%
	2,4	71.9	8.0	93.2%
6	1,4,5	105.8	10.7	94.1%
	2,3	71.7	7.2	93.2%
7	2,3,4	108.0	9.2	96.7%
	1,5	69.5	7.3	92.6%
8	2,3,5	106.7	9.5	95.6%
	1,4	70.7	8.9	89.5%
9	2,4,5	107.0	10.5	95.2%
	1,3	70.4	7.6	90.5%
10	3,4,5	107.4	10.1	95.1%
	1,2	70.0	7.3	93.0%

## Data Availability

No new data were created or analyzed in this study. Data sharing is not applicable to this article.
